# Draft genome sequence of *Mesotoga* strain PhosAC3, a mesophilic member of the bacterial order *Thermotogales*, isolated from a digestor treating phosphogypsum in Tunisia

**DOI:** 10.1186/1944-3277-10-12

**Published:** 2015-05-01

**Authors:** Wajdi Ben Hania, Khaled Fadhlaoui, Céline Brochier-Armanet, Cécile Persillon, Anne Postec, Moktar Hamdi, Alain Dolla, Bernard Ollivier, Marie-Laure Fardeau, Jean Le Mer, Gaël Erauso

**Affiliations:** Aix-Marseille Université, Université du Sud Toulon-Var, CNRS/INSU, IRD, Mediterranean Institute of Oceanography (MIO), UM 110, F-13288 Marseille, cedex 09, France; Laboratoire d’Ecologie et de Technologie Microbienne, Institut National des Sciences Appliquées et de Technologie, Faculté des Sciences de Carthage, Centre Urbain Nord, BP 676, Tunis, 1080 Tunisia; CNRS, UMR 5558, Laboratoire de Biométrie et Biologie Evolutive, Université de Lyon, Université Lyon 1, 43 boulevard du 11 novembre 1918, Villeurbanne, F-69622 France; Protéus SA, 70 Allée Graham Bell, Nîmes, F-30035 France; Aix-Marseille Université, CNRS, LCB-UMR7283, Marseille, F-13009 France

**Keywords:** Anaerobic, Mesophilic, *Thermotogales*, *Mesotoga*

## Abstract

*Mesotoga* strain PhosAc3 was the first mesophilic cultivated member of the order *Thermotogales.* This genus currently contain two described species, *M. prima* and *M. infera*. Strain PhosAc3, isolated from a Tunisian digestor treating phosphogypsum, is phylogenetically closely related to *M. prima* strain MesG1.Ag.4.2^T^. Strain PhosAc3 has a genome of 3.1 Mb with a G+C content of 45.2%. It contains 3,051 protein-coding genes of which 74.6% have their best reciprocal BLAST hit in the genome of the type species, strain MesG1.Ag.4.2^T^. For this reason we propose to assign strain PhosAc3 as a novel ecotype of the *Mesotoga prima* species. However, in contrast with the *M. prima* type strain, (i) it does not ferment sugars but uses them only in the presence of elemental sulfur as terminal electron acceptor, (ii) it produces only acetate and CO_2_ from sugars, whereas strain MesG1.Ag.4.2^T^ produces acetate, butyrate, isobutyrate, isovalerate, 2-methyl-butyrate and (iii) sulfides are also end products of the elemental sulfur reduction in theses growth conditions.

**Electronic supplementary material:**

The online version of this article (doi:10.1186/1944-3277-10-12) contains supplementary material, which is available to authorized users.

## Introduction

Members of the order Thermotogales typically possess a sheath-like structure called a “toga” and are mostly known as thermophilic or hyperthermophilic bacteria. Most species within this order have been isolated from heated sub-seafloors, marine hydrothermal vents, terrestrial hot springs and oil field reservoirs. Interestingly, SSU rRNA genes of Thermotogales were also detected in samples from polluted environments such as sediments of harbors and sludge from waste water treatment plants [1]. Accordingly they were also found in mesothermic enrichment cultures, notably those capable of (i) reductively dechlorinating 2, 3, 4, 5-tetrachlorobiphenyl, (ii) oxidizing hydrocarbons [2]. We reported in 2011 the first cultivation and a preliminary description of a mesophilic bacterium pertaining to the Thermotogales (strain PhosAc3) which was tentatively named “*Mesotoga sulfurireducens*” [3]. This mesophilic isolate was shown to belong to a large group of uncultivated bacteria that is distantly related to the thermophilic genus Kosmotoga. Soon after, M. prima strain MesG1.Ag.4.2^T^ isolated from sediments from Baltimore Harbor [4] and M. infera strain VNs100^T^ isolated from a water sample collected in the area of an underground gas storage [5] were fully characterized and described as new species. Strain PhosAc3 was isolated from a digestor treating phosphogypsum inoculated with a mixture of marine sediments and sludge originating from a dump and a wastewater treatment plant in Tunisia. It grows at temperatures between 30°C and 50°C (optimum 40°C) and uses fructose and lactate as energy sources. Phylogenetic analyses based on 16S rRNA gene sequences revealed that strain PhosAc3 is closely related to M. prima strain MesG1.Ag.4.2^T^[3].

Here we report on further taxonomic and physiological studies on strain PhosAc3 and describe the draft genome sequence and its annotation. We show that while they belong to the same species, PhosAc3 and MesG1.Ag.4.2^T^ strains exhibit significant phenotypic and metabolic differences and that their genomes differ by about 25% in gene content.

### Organism information

#### Classification and features

**Figure 1:**
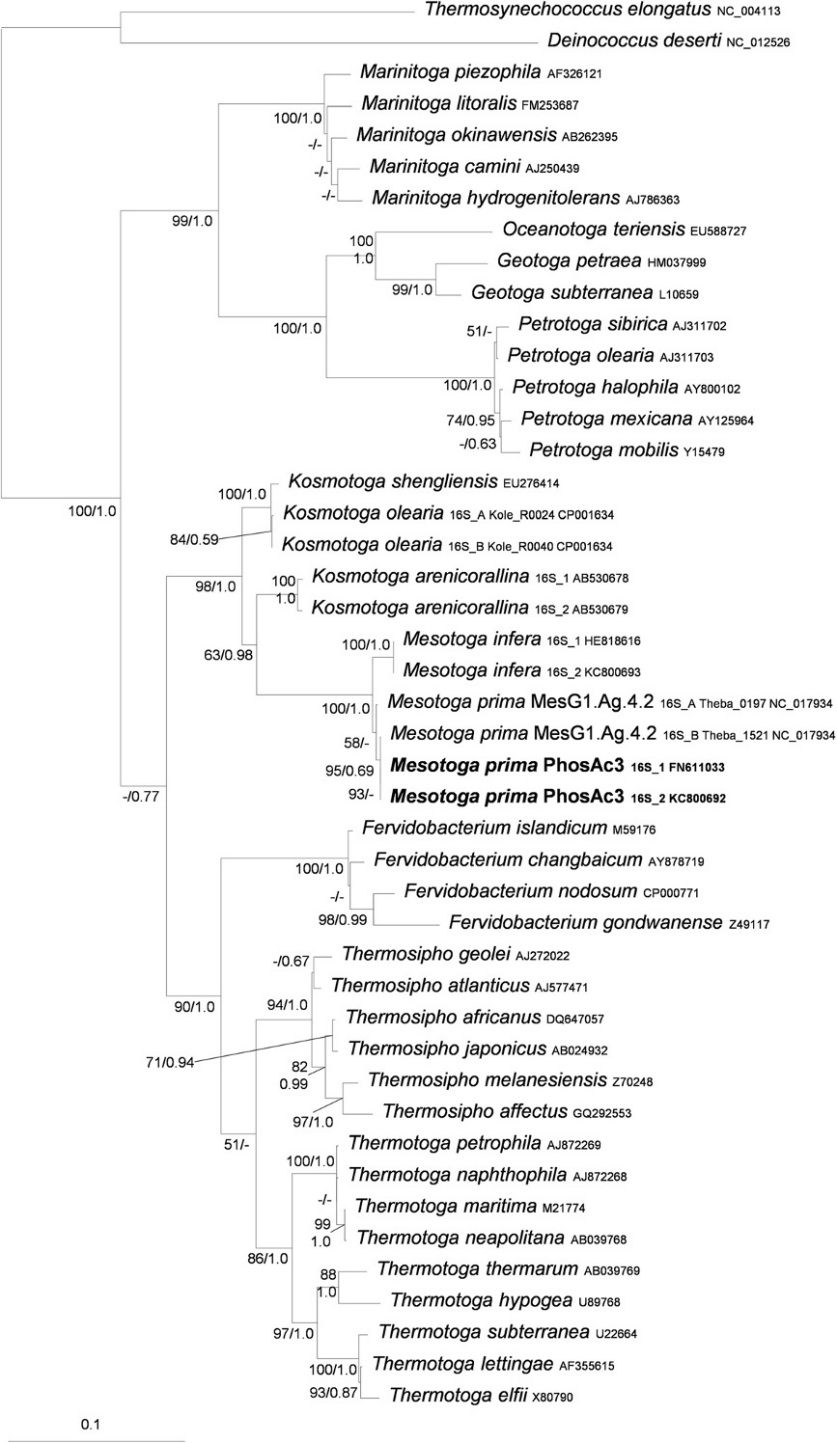
**Rooted maximum likelihood phylogeny of SSU rRNA sequences from** ***Thermotogales*** **(45 sequences, 1265 nucleotide positions).** Numbers at nodes represent the bootstrap values estimated by the non-parametric bootstrap procedure implemented in Treefinder (100 replicates of the original alignments) and the posterior probabilities computed by Mr Bayes (only values greater than 50% and 0.5 respectively, are shown). The scale bar represents the average number of substitution per site.

Genomic sequences of strain PhosAc3 showed that it possesses two copies of the 16S rRNA gene. As for M. infera, the two 16S rRNA coding genes found in PhosAc3 are 100% identical (this was further confirmed by re-sequencing of PCR products obtained using two primers pairs specifically designed to target the two 16S rRNA gene loci respectively). This situation contrasts with that of MesG1.Ag.4.2^T^, which was reported to harbor two distinct 16S rRNA genes that are 99.1% identical (Theba_0197 and Theba_1521). The two 16S rRNA genes of strain PhosAc3 share 99.2% identity with the sequence of MesG1Ag4.2. 16S rRNA gene A*.* Experiments conducted by the DSMZ (Deutsche Sammlung von Mikroorganismen und Zellkulturen GmbH) Identification Service on PhosAc3 DNA revealed 78.7% of DNA-DNA re-association with M. prima MesG1.Ag.4.2^T^, which is a sufficient criterion to classify both strain in the same species. The phylogenetic position of strain PhosAc3 is shown in Figure 1.

**Figure 2:**
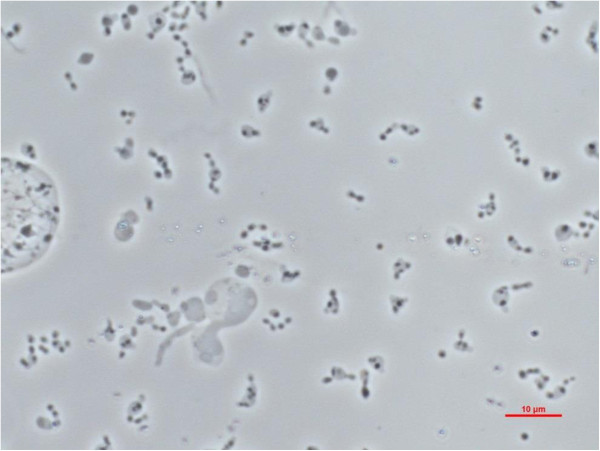
**Phase contrast micrographs of strain PhosAc3.** Scale bar: 10 μm.

Strain PhosAc3 is a Gram-negative, pleomorphic bacterium. Cells appeared mostly as chains with a rod to coccoid shape of 2–4 μm long and 1–2 μm in diameter (Figure 2). They were non-motile. Strain PhosAc3 is a strict anaerobe. It is a mesophilic bacterium with an optimal growth temperature at 40°C (range 30-50°C). Additional analyses were performed to complete the characterisation of strain PhosAc3 using the same experimental procedures as detailed previously [3, 4]. The optimal growth NaCl concentration was found at 2 g. L^−1^ (range 0–30 g. L^−1^). The optimum pH range for growth was 6.9 (range 6.7-7.9). Elemental sulfur (10 g. L^−1^) was used as terminal electron acceptor, but not thiosulfate, sulfate or sulfite. Strain PhosAc3 used poorly yeast extract but requires it at low concentration (at 0.5 g. L^−1^) to grow on sugars, peptides and organic acids (arabinose, fructose, glucose, maltose, mannose, raffinose, saccharose, xylose, cellobiose, peptone, lactate and pyruvate) probably as vitamins and other growth factor sources. In contrast, the following substrates were not utilized: galactose, lactose, ribose, gelatin, casein, xylan, cellulose, acetate, butyrate, fumarate, succinate, ethanol, methanol, 1-propanol, and propionate. No growth by fermentation was observed with any combination of yeast extract and peptides or sugars in the absence of elemental sulfur, contrasting with what was reported for M. prima strain MesG1Ag4.2 (Additional file 1: Table S1). Surprisingly, acetate was also required at low concentration (2 mM) to initiate growth most likely to serve as carbon source for anabolism and thus was latter systematically added to the culture medium. End products of sugar metabolism were acetate and CO_2_. Sulfide production resulted from reduction of elemental sulfur. In any conditions of cultures, hydrogen was detected only as traces with concentrations around 1 μM measured in the gas phase. Finally, no growth was detected with H_2_/CO_2_ gas (200 kPa) in the headspace, with or without acetate added to the culture medium.

All these informations on strain PhosAc3 are summarized in Table 1.

**Figure 3:**
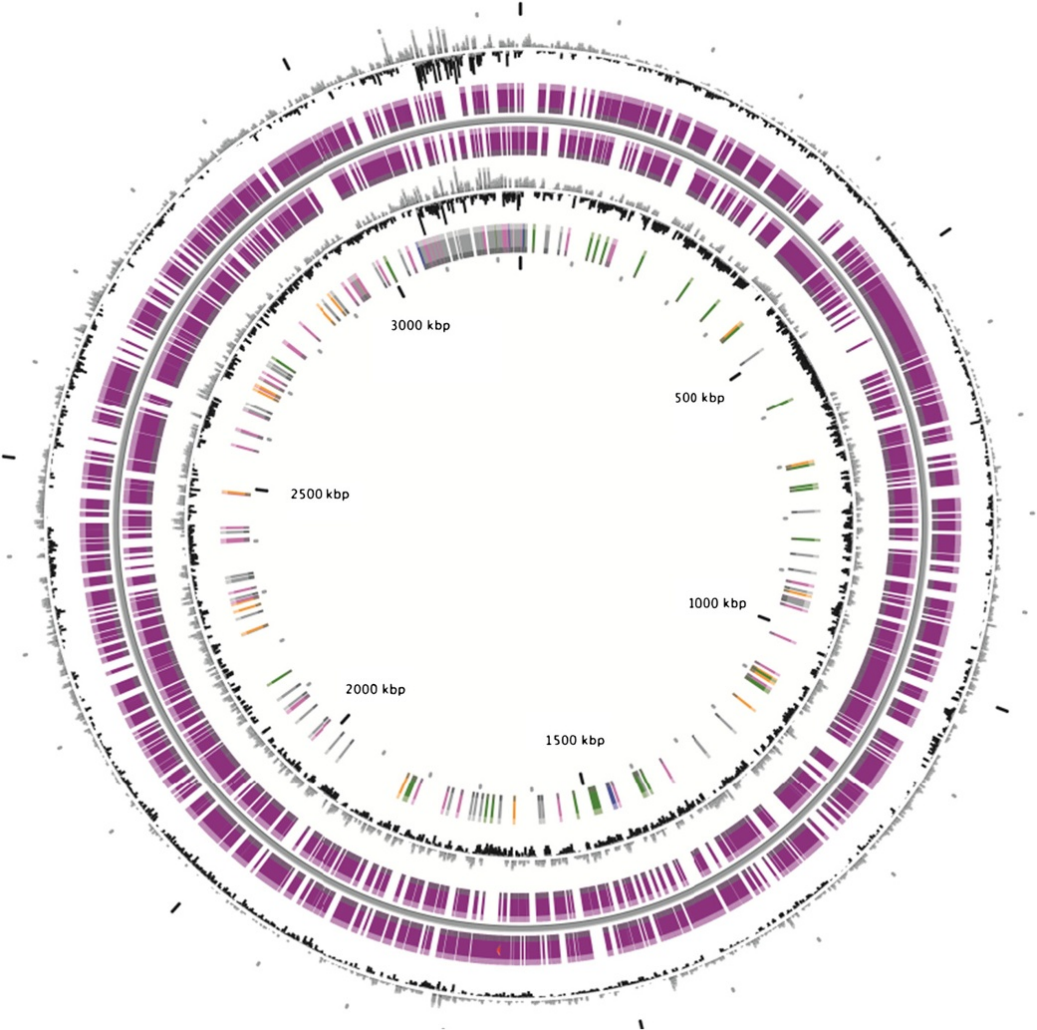
**Circular representation of the** ***Mesotoga*** **strain PhosAc3 chromosome.** Circles display (from the outside): (1) GC percent deviation (GC window - mean GC) in a 1000-bp window, (2) Predicted CDSs transcribed in the clockwise direction, (3) Predicted CDSs transcribed in the counterclockwise direction. Genes displayed in (2) and (3) are color-coded according different categories. Red and blue: MaGe validated annotations, orange: MicroScope automatic annotation with a reference genome, purple: Primary/Automatic annotations. (4) GC skew (G+C/G-C) in a 1000-bp window. (5) rRNA (blue), tRNA (green), misc_RNA (orange), Transposable elements (pink) and pseudogenes (grey).

#### Chemotaxonomic data

The fatty acid analysis was performed by the DSMZ on a PhosAc3 culture stopped at the end of exponential phase. Fatty acids were extracted using the method of Miller [12], analyzed by gas chromatography (gas chromatograph, model 6890 N, Agilent Technologies) and the resulting profile was determined using the Microbial Identification System (MIDI, Sherlock Version 6.1; database, TSBA40). The fatty acid pattern of strain Phos Ac3 was similar to that of *M. infera* (Additional file 1: Table S2). In contrast to these bacteria, C14 was not detected in M. prima type species (MesG1.Ag.4.2^T^) thus suggesting that strain PhosAc3 should be considered as novel ecotype of M. prima species.

### Genome sequencing information

#### Genome project history

This organism was selected for sequencing on the basis of its environmental and biotechnological relevance to issues in global carbon cycling, bioremediation of polluted soils and its significance in studying the evolutionary mechanisms of adaptation to moderate temperatures [13]. The genome project and an improved-high-quality-draft genome sequence have been deposited in the GOLD and IMG databases respectively. A summary of the project information is shown in Table 2.

**Table 2:** **Project information**

MIGS ID	Property	Term
MIGS 31	Finishing quality	High-quality draft
MIGS 28	Libraries used	454 paired-end 8- kb non-cloned libraries
MIGS 29	Sequencing platform	454 GS FLX Titanium
MIGS 31.2	Fold coverage	49×
MIGS 30	Assemblers	Celera
MIGS 32	Gene calling method	AmiGene 2.0 and IMG/ER
	Locus Tag	PHOSAC3
	Genbank ID	CARH01000001 to CARH01000099
	Genbank date of release	April 10, 2013
	GOLD ID	Gp0041593
	BIOPROJECT	
	Project relevance	Evolution (thermophily/mesophily), Bioremediation
MIGS 13	Source material identifier	DSM 24444

**Table 1:** **Classification and general features of** ***Mesotoga*** **strain PhosAc3**

MIGS ID	Property	Term	Evidence code ^a^
	Current classification	Domain *Bacteria*	TAS [6]
		Phylum *Thermotogae*	TAS [7]
		Class *Thermotogae*	TAS [7, 8]
		Order *Thermotogales*	TAS [7, 9]
		Family *Thermotogaceae*	TAS [7, 10]
		Genus *Mesotoga*	TAS [4]
		Species *Mesotoga prima*	IDA
		PhosAc3	TAS [3]
	Gram stain	Gram - negative	IDA
	Cell shape	Rod to coccoid with spheroids	TAS [3]
	Motility	Non-motile	IDA
	Sporulation	Non-sporulating	IDA
	Temperature range	30°C to 50°C	TAS [3]
	Optimum temperature	40°C	IDA
	pH range; Optimum		
	Carbon source	Acetate as carbon source, Sugars (pentoses and hexoses) and small organic acids (e.g. lactate, pyruvate) as energy sources	TAS [3]
MIGS-6	Habitat	Mesothermic anaerobic reactor	TAS [3]
MIGS-6.3	Salinity	0 to 3% (optimum 0.2%)	IDA
MIGS-22	Oxygen	Anaerobic	TAS [3]
MIGS-15	Biotic relationship	Free living	TAS [3]
MIGS-14	Pathogenicity	Non-pathogen	NAS
MIGS-4	Geographic location	Tunis - Tunisia	TAS [3]
MIGS-5	Sample collection time	2008	IDA
MIGS-4.1 MIGS-4.2	Latitude	32.38639	IDA
	Longitude	11.45833	IDA
MIGS-4.4	Altitude	3 meters	IDA

^a^Evidence codes - IDA: Inferred from Direct Assay; TAS: Traceable Author Statement (i.e., a direct report exists in the literature); NAS: Non-traceable Author Statement (i.e., not directly observed for the living, isolated sample, but based on a generally accepted property for the species, or anecdotal evidence). These evidence codes are from of the Gene Ontology project [11].

**Table 3:** **Genome statistics for** ***M. prima*** **strain PhosAc3**

Attribute	Value
Genome size^a^ (bp)	3,113,612
DNA coding region (bp)	2,646,577
DNA G+C content (bp)	1,407,041
DNA scaffolds	130
Total genes	3,123
Protein-coding genes	3,051
RNA genes^b^	72
Pseudo genes	78
Genes in internal clusters	ND
Genes with function prediction	2,115
Genes assigned to COGs	1758
Genes assigned Pfam domains	2,173
Genes with signal peptides	77
Genes with transmembrane helices	813
CRISPR repeats	9

^a^Or 3,243,715 bp without undertermined pb. ^b^Non-coding RNA =17. ^c^rRNA=4 copies of 5S, 2 copies of 16S and 3 copies of 23S rRNA, tRNA=47. ND: not determined.

**Table 4:** **Number of genes associated with general COG functional categories**

Code	Value	% age	Description
J	133	7.01	Translation, ribosomal structure and biogenesis
A	1	0.03	RNA processing and modification
K	102	5.37	Transcription
L	114	6.01	Replication, recombination and repair
B	2	0.07	Chromatin structure and dynamics
D	15	0.79	Cell cycle control, cell division, chromosome partitioning
V	39	2.05	Defense mechanisms
T	52	2.74	Signal transduction mechanisms
M	92	4.85	Cell wall/membrane/envelope biogenesis
N	6	0.32	Cell motility*
U	20	1.05	Intracellular trafficking, secretion, and vesicular
C	117	6.16	Energy production and conversion
G	243	12.80	Carbohydrate transport and metabolism
E	229	12.07	Amino acid transport and metabolism
F	57	3	Nucleotide transport and metabolism
H	48	2.53	Coenzyme transport and metabolism
I	43	2.27	Lipid transport and metabolism
P	119	6.27	Inorganic ion transport and metabolism
Q	22	1.16	Secondary metabolites biosynthesis, transport and catabolism
R	254	12.91	General function prediction only
S	141	7.43	Function unknown
J	133	7.01	Translation, ribosomal structure and biogenesis
-	1365	43.71	Not in COG

The total is based on the total number of protein coding genes in the annotated genome. *Cell motility COG categories may also includes genes involved in secretion systems such as TSS2. This can explain the occurrence of genes of this category in the genome of strain PhosAc3 whilst this bacterium is non-motile.

#### Growth conditions and DNA isolation

Genomic DNA was isolated from an exponentially growing culture of strain PhosAc3 using the protocol of Marteinsson et al. [14].

#### Genome sequencing and assembly

*De novo* whole-genome shotgun sequencing was performed by combining a single and a long paired end (8 kbp) non-cloned libraries sequencing using the Roche Titanium pyrosequencing GS FLX+ technology (MWG Eurofins). This produced 350,813 reads with an average length of 439 bp for a total number of sequenced bases of 154,143,916 representing a sequencing depth of 49×. Using Celera Assembler software (v.6.1) both data sets could be assembled into four scaffolds including 14 large contigs (>1,000 bp) and 127 small contigs.

#### Genome annotation

Gene predictions annotation and comparative genomic analyses were performed using the MicroScope annotation platform [15]. The predicted CDSs were translated and used to search the National Center for Biotechnology Information non-redundant database, UniProt, TIGRFam, Pfam, PRIAM, KEGG, COGs, and InterPro. These data sources were combined to assert a product description for each predicted protein. Non-coding genes and miscellaneous features were predicted using tRNAscan-SE, RNAMMer, Rfam, TMHMM, and signalP. Additional gene prediction analysis and functional annotation were performed within the Integrated Microbial Genomes Expert Review platform [16]. CRISPR were searched using CRISPRFinder [17]. Table 3 presents the project information and its association with MIGS version 2.0 compliance [18].

### Genome properties

The overall genome size estimated for M. prima strain PhosAc3 is 3,113,612 bp, significantly larger than that of the M. prima type strain MesG1.Ag.4.2 (2,974,229 bp) [19] and is composed of a unique circular chromosome (no plasmid was found in contrast to MesG1.Ag.4.2^T^). The average genome G+C content of strain PhosAc3 of 45.19% is close to that of MesG1.Ag.4.2^T^ (45.45%). It contains two ribosomal operons, 47 tRNAs and 3,051 predicted protein-coding genes (Table 3; Figure 3).

### Insights from the genome sequence

Like *Mesotoga prima* (strain MesG1.Ag.4.2^T^), Mesotoga strain PhosAc3 possesses a significantly larger genome (3.11 and 2.97 Mb respectively) than their thermophilic counterparts within the Thermotogales whose genome size ranged from 1.86 to 2.30 Mb. Of the 3051 protein encoding genes (CDS) of strain PhosAc3, 2392 (78.4%) have their best homologs (satisfying the bi-directional best hit criterion) in the complete genome of M. prima and are clustered in 273 syntons (cluster of at least two contiguous genes) in the two strains (SM Figure 1). For comparison sake, the two Mesotoga strains, MesG1.Ag.4.2^T^ and PhosAc3, share respectively 1468 and 1542 CDS with the closely related species Kosmotoga olearia strain TBF 19.5.1 (SM Figure 1). It seems that the supplementary genes found in Mesotoga strain PhosAc3 (not present in K. olearia) have been acquired by LGT mostly from mesophilic members of the Firmicutes (peculiarly within the Clostridiales order) to the Mesotoga (data not shown) with whom they share the same microbial habitat [19]. As previously observed for Mesotoga*prima* strain MesG1.Ag.4.2^T^, the largest fractions of the genes presumably acquired by LGT are involved in amino acids transport and metabolism (COG category E), secondary metabolite biosynthesis (COG category Q) and signal transduction mechanisms (COG category T) (Table 4; data not shown). Of the 659 genes of Mesotoga strain PhosAc3 with no detectable homologs in M. prima strain MesG1.Ag.4.2^T^, the majority (65%) are annotated as unknown function proteins, 58 (8,8%) correspond to transposons and the rest to poorly characterized functions (COG categories S and R).

## Conclusion

Based on taxonomic and genomic criteria, Mesotoga strain PhosAc3 should be considered a novel strain of the M. prima species. Besides numerous similarities, both strains exhibit clear differences in their phenotypic features and even to their gene content. They may therefore represent distinct ecotypes as previously defined [20, 21]. Strain PhosAc3, like M. infera, is capable of significant growth on simple substrates (sugars and organic acids) only in the presence of elemental sulfur as terminal electron acceptor suggesting that these substrates are oxidized rather than fermented. This sharply contrasts with the reported fermentative metabolism of sugars of M. prima type species (strain MesG1.Ag.4.2^T^). Other differences between strain PhosAc3 and M. prima strain MesG1.Ag.4.2^T^ include the end products of sugar metabolism, the optimum NaCl concentration for growth and the range of electron acceptors used (Table 1). The availability of the genome sequences of two Mesotoga strains offers a good opportunity to look in further details the genomic determinants that may be responsible of the metabolic differences observed between the two strains. Moreover, the comparison with other Thermotogales genomes should bring relevant information regarding the bacterial adaptation to novel ecological niches (from hot to mesothermic biotopes) and the importance of lateral gene transfer in such evolutionary processes [13].

## Electronic supplementary material

Additional file 1: Table S1.: Differential characteristics between *Mesotoga prima* strains and *M. infera*. **Table S2.** Fatty acids composition of *Mesotoga prima* strains and *M. infera*. **Figure S1.** Number of common (core) or strain-specific genes in *Mesotoga* strains and *Kosmotoga olearia* TBF 19.5.1. (DOCX 123 KB)
