# Supplementary material for: Draft genome sequence of Mesotoga strain PhosAC3, a mesophilic member of the bacterial order Thermotogales, isolated from a digestor treating phosphogypsum in Tunisia
Source: Stand Genomic Sci. 2015 May 1;10:12. doi: 10.1186/1944-3277-10-12 (PMC4603820; doi:10.1186/1944-3277-10-12)
Supplement: Supplementary file 1 — Additional file 1: Table S1.: Differential characteristics between Mesotoga prima strains and M. infera. Table S2. Fatty acids composition of Mesotoga prima strains and M. infera. Figure S1. Number of common (core) or strain-specific genes in Mesotoga strains and Kosmotoga olearia TBF 19.5.1. (DOCX 123 KB) [file 40793_2014_945_MOESM1_ESM.docx]

**Table S1.** Differential characteristics between *Mesotoga prima* strains and *M. infera*

| **Characteristics** | ***Mesotoga prima* strain PhosAc3** | ***Mesotoga prima* MesG1.Ag.4.2^T^** | ***Mesotoga infera* VNs100^T^** |
| --- | --- | --- | --- |
| Source | Tunisian mesothermic digester | Sediments of Baltimore Harbour | Deep aquifer, France |
| Electron acceptors: |  |  |  |
| Thiosulfate | - | +/- | - |
| Sulfite | - | +/- | - |
| Elemental sulfur | + | +/- | + |
| Sulfate | **-** | - | - |
| Temperature range | 30-50 [40] | 20-50 [37] | 30-50 [45] |
| NaCl range % | 0.-3.0 [0.2] | 2.0-6.0 [4.0] | 0-1.5 [0.2] |
| G+C content (%) | 45.3 | 45.3 | 47.5 |
| Substrates: |  |  |  |
| peptone | + | + | ND |
| arabinose | + | +/- | + |
| fructose | + | + | + |
| glucose | + | +/- | + |
| maltose | + | + | + |
| mannose | + | + | + |
| raffinose | + | ND | + |
| saccharose | + | + | + |
| xylose | + | + | + |
| cellobiose | + | +/- | + |
| lactate | + | ND | + |
| pyruvate | + | +/- | ND |
| galactose | - | + | + |
| lactose | - | + | + |
| ribose | - | + | + |
| casein | - | +/- | ND |
| End products of sugar metabolism | Acetate, CO_2_ | Acetate, butyrate, isobutyrate, isovalerate, 2-methyl-butyrate | Acetate, CO_2_ |

**Table S2: Fatty acids composition of** [***Mesotoga***](http://dx.doi.org/10.1601/nm.23646) [***prima***](http://dx.doi.org/10.1601/nm.23647) **strains and** [***M. infera***](http://dx.doi.org/10.1601/nm.24427)

| Fatty acids | [*M. prima*](http://dx.doi.org/10.1601/nm.23647)  PhosAc3 | [*M. prima*](http://dx.doi.org/10.1601/nm.23647)  MesG1.Ag.4.2^T^ | [*M. infera*](http://dx.doi.org/10.1601/nm.24427)  VNs100^T^ |
| --- | --- | --- | --- |
| C_14:0_  C_16:0_  C _18:0_  C _18:1_^ω9c^ | 12.3  70.6  10.7  6.4 | -  37.6  23.9  26.4 | 17.2  51.1  3.8  3.8 |

**Fig.S1.** Number of common (core) or strain-specific genes in [*Mesotoga*](http://dx.doi.org/10.1601/nm.23647) strains and [*Kosmotoga olearia*](http://doi.namesforlife.com/10.1601/nm.14942) TBF 19.5.1
